# Personalised and Collaborative Learning Experience (PCLE) Framework for AI-driven Learning Management System (LMS)

**DOI:** 10.12688/f1000research.166248.1

**Published:** 2025-08-20

**Authors:** Claireta Tang Weiling, Lew Sook Ling, Ooi Shih Yin

**Affiliations:** 1 Faculty of Information Science and Technology, Multimedia University, Bukit Beruang, Melaka, 75450, Malaysia; 2 Centre for Advanced Analytics, CoE for Artificial Intelligence, Faculty of Information Science and Technology, Multimedia University, Bukit Beruang, Melaka, 75450, Malaysia

**Keywords:** Collaborative Filtering, K-Nearest Neighbours Model (KNN), Learning Management System, Neural Collaborative Filtering Model (NCF), Personalised Learning, Singular Value Decomposition Model (SVD)

## Abstract

**Background:**

Understanding student engagement and academic performance is crucial in AI-driven e-learning environments. Many learning management systems (LMS) lack effective collaborative course recommendation strategies, limiting support for personalised learning experiences.

**Methods:**

This study developed and evaluated collaborative filtering and machine learning models to generate course recommendations. Machine learning models such as K-Nearest Neighbours (KNN), Singular Value Decomposition (SVD), and Neural Collaborative Filtering (NCF) were applied. Two education-related datasets from Kaggle were used. The first contains 100,000 course reviews from Coursera, and the second dataset includes 209,000 course details and comments from Udemy. Data preprocessing was conducted to clean and structure both datasets. The model effectiveness was evaluated using Mean Absolute Error (MAE), Hit Rate (HR), and Average Reciprocal Hit Ranking (ARHR).

**Results:**

K-Nearest Neighbours showed the highest performance on the Coursera dataset, while Singular Value Decomposition and Neural Collaborative Filtering maintained stable predictive accuracy across both datasets. The findings indicate that dataset characteristics influenced model performance. K-Nearest Neighbours worked effectively with structured and consistent data, while Singular Value Decomposition and Neural Collaborative Filtering produced consistent outcomes across diverse datasets.

**Conclusions:**

This study contributes to e-learning research by demonstrating the potential of collaborative filtering and machine learning in enhancing course recommendations and promoting engagement in the learning management system. Limitations include the use of two datasets and a limited set of machine learning models. Future work aims to integrate learning styles and evaluate the framework across more diverse educational contexts to support adaptive and collaborative learning.

## 1. Introduction

### 1.1 Overview

In today’s digital age, online learning systems are becoming essential, especially highlighted by the challenges posed during the COVID-19 pandemic ( Khalaf et al., 2022). As schools and universities shifted online, the need for effective learning management systems soared. These systems are designed to provide adaptive and personalised learning experiences, helping students engage more deeply in education and succeed academically. A new machine learning-based approach ( Ayouni et al., 2021) has demonstrated great potential in enhancing student engagement in online environments by leveraging adaptive learning mechanisms and recommendation systems.

Education is no longer limited to the traditional classroom and has slowly begun to adopt technology to enhance the learning experience, offering more flexible and accessible ways to engage with content. In this context, personalised learning experiences have emerged as a vital component of effective online education. Personalisation refers to tailoring educational content and approaches to meet individual learners’ needs, preferences, and learning styles. With the increasing diversity in learning preferences, understanding and integrating these differences into e-learning platforms is crucial for fostering student engagement and success. The intersection of Artificial Intelligence (AI) and personalised learning offers promising avenues for enhancing the educational experience ( Kanchon et al., 2024), particularly in terms of adaptive learning and predictive analytics. The study by Essa et al. (2023) has highlighted the effectiveness of personalised adaptive learning technologies based on machine learning techniques in identifying student learning styles, allowing for a more tailored educational experience. The integration of AI-driven tools into e-learning, such as mobile interactive systems ( Lai et al., 2021b) and freehand writing applications ( Lai et al., 2022), has been shown to improve student engagement and learning outcomes, especially in virtual classroom settings. In addition, collaborative learning strategies have demonstrated significant benefits in virtual higher education environments ( Herrera-Pavo, 2021), enhancing knowledge sharing and peer interactions.

As AI-driven learning systems continue to evolve, the broader impact of digital transformation on education must be considered. The digital transformation of education has introduced a range of tools and methods that enhance learning experiences through personalisation, interactivity and collaboration. The study by Manzoor et al. (2022) proposed a pre-protocol to explore how mobile virtual reality (VR) influences learner motivation and engagement across different VARK (Visual, Auditory, Read/Write and Kinesthetic) learning styles, highlighting the potential of immersive technology to tailor content based on sensory preferences. A framework introduced by Haw et al. (2021) incorporates ontology-based data enrichment into a hybrid recommender system, allowing more accurate and context-aware interpretation of learner needs. The study by Nain et al. (2025) presents a bibliometric analysis of AI and teacher collaboration in educational research, offering insights into how AI tools can support collaborative teaching practices and shape future educational strategies. In addition, the study by Ong et al. (2021) propose a Neural Matrix Factorization++ model for recommendation systems, which enhances prediction accuracy by combining deep learning techniques with collaborative filtering to support more dynamic and personalised learner experiences. Furthermore, the research by Hemingway et al. (2015) highlights key factors in digital collaborative learning, such as meaningful peer interaction and structured support, which are essential in designing effective online environments. Addressing the challenges of remote teaching, Lai et al. (2022) examine a synchronous display and whiteboard-like freehand writing app that replicates classroom interactivity and supports real-time communication. Similarly, an interactive learning system introduced by Rahman et al. (2022) demonstrates how live engagement tools can strengthen conceptual understanding and participation in subjects like calculus. As a whole, these studies reflect a growing emphasis on improving online education through adaptive, interactive and collaborative approaches, paving the way for more engaging and effective virtual learning experiences.

Despite the growing recognition of personalised learning, many existing systems lack the ability to effectively predict and accommodate individual learning styles. This study aims to address this gap by developing and evaluating a Personalised and Collaborative Learning Experience (PCLE) framework that integrates collaborative filtering techniques and machine learning models to enhance learning recommendations. The use of learning style frameworks has been particularly relevant in health science education ( Childs-Kean et al., 2020), offering insights into students’ preferences and engagement strategies. Additionally, frameworks such as the Information Technology Capability (ITC) framework have demonstrated potential in enhancing learning experiences and academic achievement by improving digital learning environments ( Lew & Krishnasamy, 2023). By integrating these learning styles into the framework ( Taheri et al., 2021), the study seeks to enhance student engagement and academic performance through a more tailored and adaptive learning experience. Moreover, incorporating advanced collaborative filtering techniques, such as Adaptive KNN and SVD ( Rahman, 2023), has significantly improved the accuracy and efficiency of recommendation systems in online learning platforms.

The significance of this study lies in the potential to contribute to the educational landscape in Malaysia, particularly in the post-COVID-19 era. By focusing on the development of a framework that integrates personalised learning and collaboration, this study aligns with the Malaysian government’s initiative to cultivate future-ready talent. The proposed framework not only aims to improve academic performance but also addresses the pressing need for diversified and collaborative learning environments in digital education. For example, the Technological Pedagogical and Content Knowledge (TPACK) framework has been instrumental in improving teachers’ efficiency, student performance and engagement, underscoring the importance of well-structured digital learning frameworks in education ( Lai et al., 2021a). Furthermore, the implementation of an adaptable and personalised framework for top N course recommendations has demonstrated success in online learning ( Amin et al., 2024), suggesting the need for intelligent, tailored learning experiences.

Through a comprehensive analysis of existing literature and the application of advanced machine learning techniques ( William Fernandes et al., 2023), this paper explores the effectiveness of integrating learning styles into AI-driven e-learning systems. By identifying key factors that influence student performance and engagement, the study aspires to generate actionable insights for educators and policymakers. For instance, study by Moubayed et al. (2018) examining the relationship between student engagement and performance in e-learning environments using association rule mining have provided valuable insights into how engagement patterns influence academic outcomes. Ultimately, the findings will provide a foundation for further study in the field of personalised education, highlighting the importance of adaptive learning solutions in today’s digital age.

This study seeks to advance the understanding of personalised learning experiences within the framework of AI-driven education. By integrating collaborative filtering methods and machine learning models, the study aims to evaluate effectiveness in enhancing the learning journey for diverse student populations, fostering not only academic success but also a more engaging and inclusive educational experience. Instead of explicitly using learning style models such as VARK, this study focuses on evaluating the benchmark paper’s method, which applies collaborative filtering techniques, including K-Nearest Neighbours (KNN), Singular Value Decomposition (SVD), and Neural Collaborative Filtering (NCF), using two different datasets. This approach enables a comparative analysis of recommendation techniques in personalised learning environments, providing insights into improving student engagement and academic performance.

### 1.2 Problem statement

The educational landscape has transformed significantly in the wake of the COVID-19 pandemic, leading to a surge in demand for effective online learning management systems (LMS). However, many existing LMS fall short in providing personalised learning experiences that cater to individual learners’ needs and often lack collaborative features essential for fostering meaningful interactions among students ( Jena et al., 2023). This limitation presents a major challenge for educational institutions striving to enhance academic performance and overall satisfaction in an increasingly digital world.

Moreover, understanding individual learning styles is crucial for tailoring educational experiences to meet the diverse needs of learners. Frameworks like Kolb and VARK offer valuable insights, yet a notable gap remains in effectively integrating these learning styles into collaborative environments within LMS. Most studies have primarily focused on individual styles ( Sanjabi & Ali Montazer, 2020), neglecting the potential of combining these approaches with collaborative strategies to boost engagement and performance. For instance, generation Z of healthcare students ( Shorey et al., 2021) have distinct learning preferences that require adaptive and personalised approaches to improve learning outcomes. The study by Joorabloo et al. (2020) highlights the importance of collaborative filtering in educational recommendations, showing that improved similarity prediction enhances recommendation accuracy. Exploring how learning styles, academic records and AI assistance impact personalised collaborative learning experiences is essential for addressing these gaps.

Additionally, the absence of a structured method to incorporate learning styles into personalised and collaborative experiences restricts the effectiveness of AI-driven systems. This gap limits the ability to address diverse learning needs effectively and poses challenges to achieving better academic outcomes. Furthermore, limited evaluation of student engagement and academic performance in AI-driven LMS hinders the understanding of how personalised and collaborative learning experiences impact student success. Developing and evaluating a framework to predict and enhance student engagement and academic performance will address these challenges and provide a pathway to innovative solutions. The integration of advanced collaborative filtering approaches, such as extended collaborative filtering with adaptive K-Nearest Neighbours model (KNN), Singular Value Decomposition model (SVD) ( Rahman, 2023), has been shown to enhance recommendation accuracy and personalisation in online learning. The study aims to bridge these gaps by introducing a Personalised and Collaborative Learning Experience (PCLE) framework that evaluates collaborative filtering techniques. By exploring how the PCLE framework can enhance student engagement and academic performance, this study seeks to provide a comprehensive solution to the challenges facing the current educational landscape.

### 1.3 Study objectives

The general objective of this study is to develop and evaluate a framework for Personalised and Collaborative Learning Experience (PCLE) to enhance student engagement and academic performance in AI-driven learning environments. The specific objectives are: (1) To determine the factors contributing to students’ academic performance in the context of AI-driven digital transformation. (2) To investigate the effects of a student’s learning style, academic records and intelligence agent assistance on personalised collaborative learning experience (PCLE) for students. (3) To develop a framework of PCLE for predicting and improving students’ engagement level and academic performance for students. (4) To evaluate the student’s engagement level and academic performance with the proposed PCLE framework for students. Table 1 presents the mapping between the problem statement, study objectives and expected outputs, ensuring a structured alignment of the study focus.

**Table 1.** Mapping of problem statement, study objectives and expected outputs. This table outlines the alignment between identified problems in the current learning management system (LMS), the study’s objectives, and the anticipated research outcomes.

Problem statement	Study objectives	Expected outputs
Many existing LMS fall short in providing personalised learning experiences that cater to individual learners’ needs and often lack collaborative features essential for fostering meaningful interactions among students.	To determine the factors contributing to students' academic performance in the context of AI-driven digital transformation.	The identification of key factors influencing student engagement and academic performance.
A notable gap remains in effectively integrating these learning styles into collaborative environments within LMS.	To investigate the effects of a student's learning style, academic records and intelligence agent assistance on personalised collaborative learning experience (PCLE) for students.	A deeper understanding of how learning styles, academic history, and AI-driven assistance contribute to personalised and collaborative learning.
The absence of a structured method to incorporate learning styles into personalised and collaborative experiences restricts the effectiveness of AI-driven systems.	To develop a framework of PCLE for predicting and improving students' engagement level and academic performance for students.	The development of the PCLE framework integrating learning styles and collaborative filtering to enhance engagement and learning outcomes.
Limited evaluation of student engagement and academic performance in AI-driven LMS.	To evaluate the students’ engagement level and academic performance with the proposed PCLE framework for students.	A comprehensive assessment of the PCLE framework’s effectiveness in improving engagement and academic success.

## 2. Literature review

### 2.1 Introduction to learning styles

According to Waladi et al. (2023), various learning style models, including VARK model, Honey and Mumford model, Kolb’s Experiential Learning model, Gregorc Style Delineator model as well as Dunn and Dunn model have been explored within adaptive educational environments. The integration of artificial intelligence technology has significantly advanced e-learning systems by enabling adaptive learning that personalises educational experiences according to students’ unique learning styles. However, this approach faces limitations, including the need for large datasets to make accurate predictions and challenges in implementing continuous real-time adaptation. Despite these challenges, the value of AI-driven adaptive learning lies in the ability to enhance personalised learning by dynamically adjusting to individual learning preferences. This study aims to overcome some of these challenges by evaluating AI-driven methods within the Personalised and Collaborative Learning Experience (PCLE) framework, which adapts and predicts learning preferences. This study references learning style models such as Kolb and VARK solely for conceptual discussion and does not involve the direct use or distribution of any proprietary instruments or questionnaires. Therefore, no copyright license was required.

The study by Mpwanya & Dockrat (2020) emphasised the importance of understanding students’ learning styles and adapting instructional strategies accordingly for effective learning and improved performance. Most undergraduate logistics students displayed an accommodator learning style, indicating a preference for hands-on, experiential learning activities. However, a limitation of Mpwanya’s study is the need for further validation with larger sample sizes across different institutions. Despite this, the findings provide valuable insights into the relationship between learning styles and demographic factors in logistics education, contributing to a deeper understanding of how instructional methods can be tailored to enhance student outcomes. Similarly, this study will explore the dominant learning styles among students and examine relevance within the PCLE framework, using collaborative filtering and machine learning algorithms to personalise learning pathways.

Besides that, Subagja & Rubini (2023) discussed how aligning learning materials with students’ preferred learning styles, shaped by personal and environmental factors, can enhance the knowledge acquisition process. The study by Subagja aimed to determine the dominant learning styles among science students. Results showed that 35% of the students preferred kinesthetic learning, 30% preferred visual learning, 21% preferred auditory learning, and 14% preferred reading or writing. However, Subagja’s study was limited to a single institution and focused exclusively on science subjects. Despite these constraints, the findings provide valuable insights into learning style preferences within science education, contributing to a better understanding of how educational approaches can be adjusted to meet diverse learning needs. This study will also build on such findings by exploring the role of different learning styles in the PCLE framework, ensuring that the approach is inclusive and adaptable to various student preferences.

In conclusion, various learning style models, such as the VARK, Honey and Mumford, Kolb’s Experiential Learning, Gregorc Style Delineator, and Dunn and Dunn, have been explored in adaptive educational environments to enhance personalised learning. Integrating these models with AI-driven systems further personalises education, though challenges such as data requirements and real-time adaptation persist. Rather than relying on predefined learning styles, this study leverages collaborative filtering and machine learning techniques to improve learning recommendations in AI-driven environments. By applying collaborative filtering with KNN, SVD, and NCF, this study aims to enhance personalisation through data-driven predictions, addressing limitations in existing LMS platforms and improving student engagement and academic performance.

### 2.2 Kolb Model, Felder-Silverman Model, VARK Model, Gregorc’s Mind Styles, Riding’s Cognitive Styles and Myers-Briggs Type Indicator

Understanding different learning styles is crucial for personalising education and improving student outcomes. Key models include the Kolb Model, Felder-Silverman Model, VARK Model, Gregorc’s Mind Styles, Riding’s Cognitive Styles, and the Myers-Briggs Type Indicator. These models provide valuable insights into various learning preferences, supporting the creation of adaptive educational environments tailored to individual needs. While these models provide insights into individual preferences, this study focuses on collaborative filtering-based recommendations. By applying KNN, SVD and NCF models, course recommendations are optimised based on user interactions. The performance evaluation through MAE, HR and ARHR ensures improved recommendation accuracy, contributing to more effective AI-driven learning environments. By incorporating these methods, this study improves adaptability in course recommendations, creating a more responsive and data-driven learning experience.

The study by Mwangi and Muchiri explores the learning style preferences of physiotherapy students at Kenya Medical Training College, using Honey and Mumford’s 1982 study (as cited in Mwangi & Muchiri, 2019) and Neil Fleming’s 2001 study (as cited in Mwangi & Muchiri, 2019) questionnaires to assess students’ preferred learning approaches. Data analysis revealed that the Reflector learning style was the most prevalent among the participants, with Kinesthetic learners also being common. The VARK questionnaire proved effective in identifying preferred learning styles, which can enhance the teaching and learning process. However, Mwangi’s study faced limitations, including being restricted to a single medical college with a small sample size and not considering confounding factors such as socioeconomic status, race, and culture. Overall, the findings provide valuable insights into how learning preferences impact student performance in medical education. While learning styles provide valuable insights into student engagement, this study focuses on leveraging collaborative filtering and machine learning techniques to enhance personalised recommendations in AI-driven e-learning environments.

Furthermore, the study by Zine et al. (2019) investigates the effectiveness of various learning style models in adaptive educational environments. The author evaluates Gregorc’s Mind Styles Model, Riding’s Cognitive Style Model, Myers-Briggs Type Indicator Model, Felder-Silverman Learning Styles Model, and Kolb’s Experiential Learning Theory. The findings indicate that the Felder-Silverman model is the most suitable for adaptive e-learning due to the comprehensive approach to learning styles. However, the finding’s applicability is limited to adaptive systems and does not extend to non-adaptive contexts. Zine’s study provides valuable insights for designing adaptive educational environments by emphasising popular learning styles like those in the VARK and Kolb models. Instead of relying on predefined learning styles, this study focuses on collaborative filtering and machine learning techniques to enhance e-learning recommendations within the PCLE framework. By integrating KNN, SVD, and NCF, this study aims to improve personalised learning experiences through data-driven adaptation, addressing gaps in traditional LMS approaches.

In a nutshell, learning style models such as Kolb, Felder-Silverman, VARK, Gregorc’s Mind Styles, Riding’s Cognitive Styles, and Myers-Briggs provide valuable insights into personalising education. Each model highlights different ways students process information, enabling more tailored and effective learning experiences. Despite some challenges and limitations, these models play a crucial role in enhancing educational environments and engagement. While traditional models focus on categorising learners, AI-driven approaches such as collaborative filtering and machine learning offer dynamic personalisation, adapting recommendations based on real-time learning behavior. By integrating KNN, SVD, and NCF, this study enhances personalised learning experiences through data-driven adaptation, addressing gaps in traditional LMS approaches.

### 2.3 Integration of learning styles in E-learning and adaptive systems

With the rapid advancement in technology, the application of learning styles in e-learning systems has demonstrated significant value, particularly in personalising learning paths ( Seghroucheni & Chekour, 2023). The use of the Felder-Silverman learning styles model has shown effectiveness. The results indicate that this effectiveness has come up against the technical characteristics of mobile environments. The model’s limitation is that optimal functioning occurs only under specific conditions, which can restrict adaptability to mobile platforms. Despite these constraints, the study by Seghroucheni highlights opportunities for improving the integration of learning styles into mobile learning environments and suggests potential technical enhancements to better accommodate these contexts.

The study by Glazunova et al. (2020) focuses on adapting educational content based on identified learning styles. The system effectively customises educational materials to align with individual learning preferences, showcasing significant promises in enhancing personalised learning experiences. Despite this, Glazunova’s study has a limitation due to the limited testing of the system’s scalability and the ability to adapt in real-time environments. The findings underscore the potential of data-driven learning style identification to substantially improve adaptive learning platforms. The findings offer valuable insights into the development of tailored educational approaches, providing practical implications for refining personalised educational strategies and improving overall learning outcomes. This approach aligns with the goals of this current study where data-driven methods, such as the collaborative filtering and machine learning techniques, will be incorporated into the PCLE framework to ensure more scalable and adaptive solutions.

Many current personalised educational platforms struggle to meet the diverse needs of students with varying intellectual abilities, learning paces, and preferences ( William Fernandes et al., 2023). To address this issue, the study by William employs supervised machine learning techniques to dynamically schedule assignments and educational activities based on individual student characteristics, such as proficiency level, interests, and learning preferences. Five machine learning models which are Logistic Regression (LR), K-Nearest Neighbours (KNN), Support Vector Machine (SVM), Decision Tree (DT), and Random Forest (RF) were evaluated, with Random Forest showing the highest effectiveness. Random Forest model achieved a 94% F1-score and accuracy rate, successfully classifying students into the most appropriate learning modes. William’s study makes a significant contribution to developing equitable and adaptive learning environments and provides a strong foundation for further advancements in personalised education. In this study, similar machine learning methods like KNN and SVD will be utilised within the PCLE framework to further enhance the dynamic adaptation of learning paths, ensuring that diverse student needs are met efficiently.

**Figure 1.**
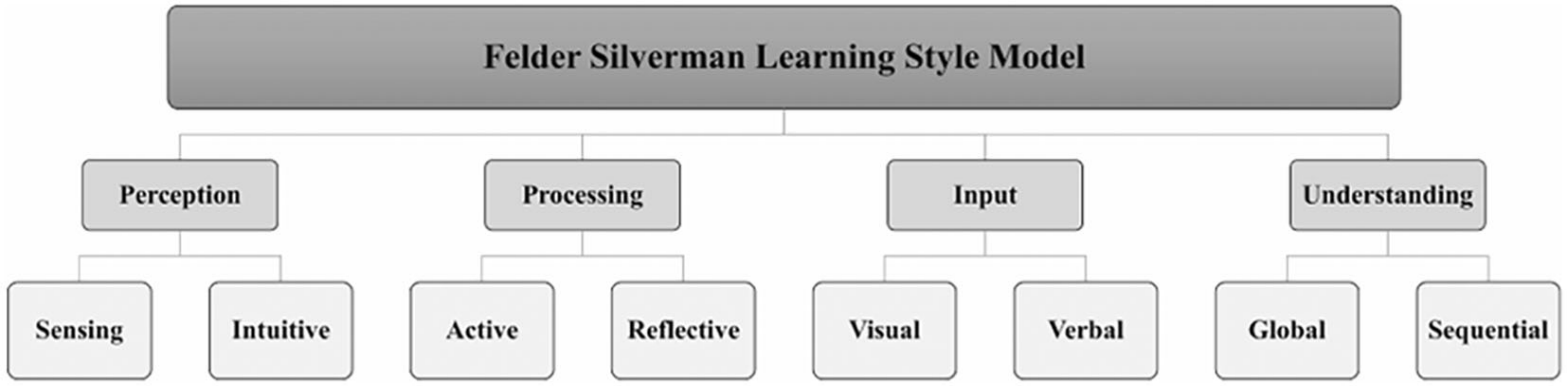
Felder and Silverman Learning Style model ( Rasheed & Wahid, 2021). An overview of the dimensions of learning preferences as defined in the Felder-Silverman model.

In summary, incorporating learning styles into e-learning and adaptive systems can significantly improve the personalisation and effectiveness of these platforms. As shown in Figure 1, the Felder-Silverman Learning Style Model ( Rasheed & Wahid, 2021) provides a structured approach to categorising learning preferences. However, limitations in adapting Felder-Silverman Learning Style Model to mobile environments must be addressed to maximise effectiveness in adaptive learning systems. This study focuses on leveraging collaborative filtering and AI-driven solutions, which offers greater flexibility for integration. Additionally, advances in machine learning also offer promising ways to better tailor education to individual needs, although more work is needed to fully meet the diverse requirements of all students. This highlights the importance of integrating collaborative filtering and machine learning into adaptive systems to improve student engagement and academic performance, as proposed in this study.

### 2.4 Impact of learning styles on academic performance and engagement

The study conducted by Subramaniam et al. (2023) investigates the prevalence of Kolb’s learning styles among students and examines the impact on learning outcomes. By using a quantitative survey method with the Kolb Learning Style questionnaire, data was collected from eighty students and analysed using descriptive statistics and linear regression. The analysis revealed that Diverger was the most common learning style among participants, followed by Assimilator, Accommodator, and Converger. Kolb’s framework categorises learners into four distinct styles: Converger, Diverger, Assimilator, and Accommodator, each representing different approaches to processing and applying knowledge. Subramaniam’s study describes Divergers as learners who engage in concrete experience and reflective observation, approaching situations from multiple perspectives. This style involves brainstorming, gathering information, and analysing different possibilities before making decisions. Assimilators prefer abstract conceptualisation and reflective observation, focusing on theoretical understanding rather than practical application. Convergers adopt abstract conceptualisation and active experimentation, excelling in problem-solving and applying theories to real-world situations, particularly in technical tasks that require logical reasoning. Accommodators, associated with concrete experience and active experimentation, learn best through hands-on activities, trial and error, and direct engagement with the environment. Although the small sample size limits the generalisability and validity of the results, the findings provide useful insights for educators to design instructional methods that accommodate different learning preferences. This highlights the need for creating learning environments that cater to different learning styles, which could help improve academic outcomes for all students.

In a related study, Kamal et al. (2021) examines learning preferences among healthcare students and the impact on academic performance. The findings reveal that most respondents prefer unimodal learning styles over multimodal ones. Despite the limitations of a small sample size and a single institution, the paper provides valuable insights into healthcare students’ learning preferences and suggests potential improvements for educational strategies.

Besides that, the study by Yean et al. (2024) investigates the preferences of learning styles among students learning a third language at a public university. Utilising an adapted version of Honey and Mumford’s and Kolb’s Learning Style questionnaires, the paper found that the Reflector style was the most popular choice among participants. However, Yean’s study does not examine how these learning styles impact language learning performance. Despite this limitation, the findings underscore the importance of tailored learning strategies for multilingual education. In contrast, this study aims to not only explore learning style preferences but also evaluate relevance in academic performance, filling the gap in previous study. By examining personalised learning strategies, this study aims to offer more actionable insights for improving student engagement and performance.

The study by Taheri examines how Kolb and VARK learning styles, along with emotional creativity, impact academic performance among dental students ( Taheri et al., 2021). Through a descriptive-analytical approach, data were collected from 250 dental students in the third semester. The results show that the accommodating learning style is the most common among the participants. Taheri’s study is limited by focusing on dental students alone and the lack of control over other influencing variables. Nonetheless, the findings offer valuable insights into the interaction between learning styles, emotional creativity, and academic success in this specialised field.

Furthermore, Rastovski & Tomić (2023) examines the VARK model’s validity and reliability in evaluating learning styles and the impact on various learning outcomes. The review of 40 articles confirms that the VARK questionnaire is widely recognised as a valid and reliable tool for assessing individual learning style preferences. However, the review also identifies a limitation in the empirical testing of the VARK questionnaire across different educational contexts. Overall, this review offers valuable insights into the strengths and limitations of the VARK model and highlights the need to consider individual differences when designing educational materials and programs.

The investigation into AI-based personalised e-learning systems reveals the effectiveness in providing tailored learning experiences compared to conventional methods ( Murtaza et al., 2022). The proposed framework offers personalised e-learning to each learner. However, a limitation is the lack of diverse user personas, which restricts understanding of varied attitudes and behaviours. Despite this limitation, the framework successfully delivers efficient, individualised learning solutions.

In conclusion, tailoring education to accommodate different learning styles can enhance both academic performance and student engagement. Aligning teaching methods with students’ preferred learning approaches often leads to better outcomes. Although personalised e-learning shows promise, challenges remain in addressing the full range of diverse learning needs. This aligns with the focus of this study, which aims to evaluate collaborative filtering in personalised, AI-driven systems to improve student engagement and academic performance. By addressing the gap in personalised learning experiences for diverse student needs, this study aims to further enhance the way education can be tailored for improved engagement and academic success.

### 2.5 Collaborative learning styles

The study by Khalaf et al. (2022) examines the effectiveness of e-learning management systems, specifically focusing on Moodle and Blackboard. Both platforms offer valuable tools for personalised and collaborative learning. Moodle is recognised for being more cost-effective in the long run and better equipped for interacting with external APIs and outdated systems, making a more flexible option compared to Blackboard. However, several limitations were noted, including management challenges, technical issues within the e-learning system, and financial constraints. Despite these challenges, the findings highlight the potential of current e-learning systems as models for enhancing student engagement and optimising the use of learning technologies, especially during times of crisis such as the COVID-19 pandemic. The integration of analytical insights in collaborative learning management systems has further demonstrated potential in improving student engagement and learning outcomes, providing a basis for enhancing existing platforms ( Lai et al., 2024). Collaborative learning tools provided by these platforms can be crucial in creating dynamic and interactive educational experiences.

In another study by Alanya-Beltran (2024) introduces a personalised learning recommendation system for e-learning platforms, employing collaborative filtering and machine learning techniques. User-based and item-based collaborative filtering approaches are applied to analyse interactions between learners and educational content, identifying patterns of similarity among students. Machine learning models, such as decision trees, are used to generate tailored recommendations based on preferences, historical behaviour, and performance indicators. The proposed autoencoder model demonstrated superior accuracy in predicting course ratings compared to other methods. However, Alanya-Beltran’s study is limited by the availability of standardised databases. Despite this, the system offers valuable opportunities for students to enhance learning through dynamic and adaptable recommendations.

Additionally, Jena et al. (2023) presents an e-Learning course recommender system using collaborative filtering models to assist learners in selecting courses based on personal preferences. The study by Jena evaluates machine intelligence-based models, including K-nearest neighbour (KNN), Singular Value Decomposition (SVD), and neural network–based collaborative filtering (NCF), on a dataset of one lakh Coursera course reviews from Kaggle. The results show that KNN outperforms other models by achieving higher hit rates (HR), average reciprocal hit ranking (ARHR), and lower mean absolute error (MAE). Despite the difficulty in detecting small variations within customer segments, the system effectively aids learners in making informed course selections tailored to individual preferences.

**Figure 2.**
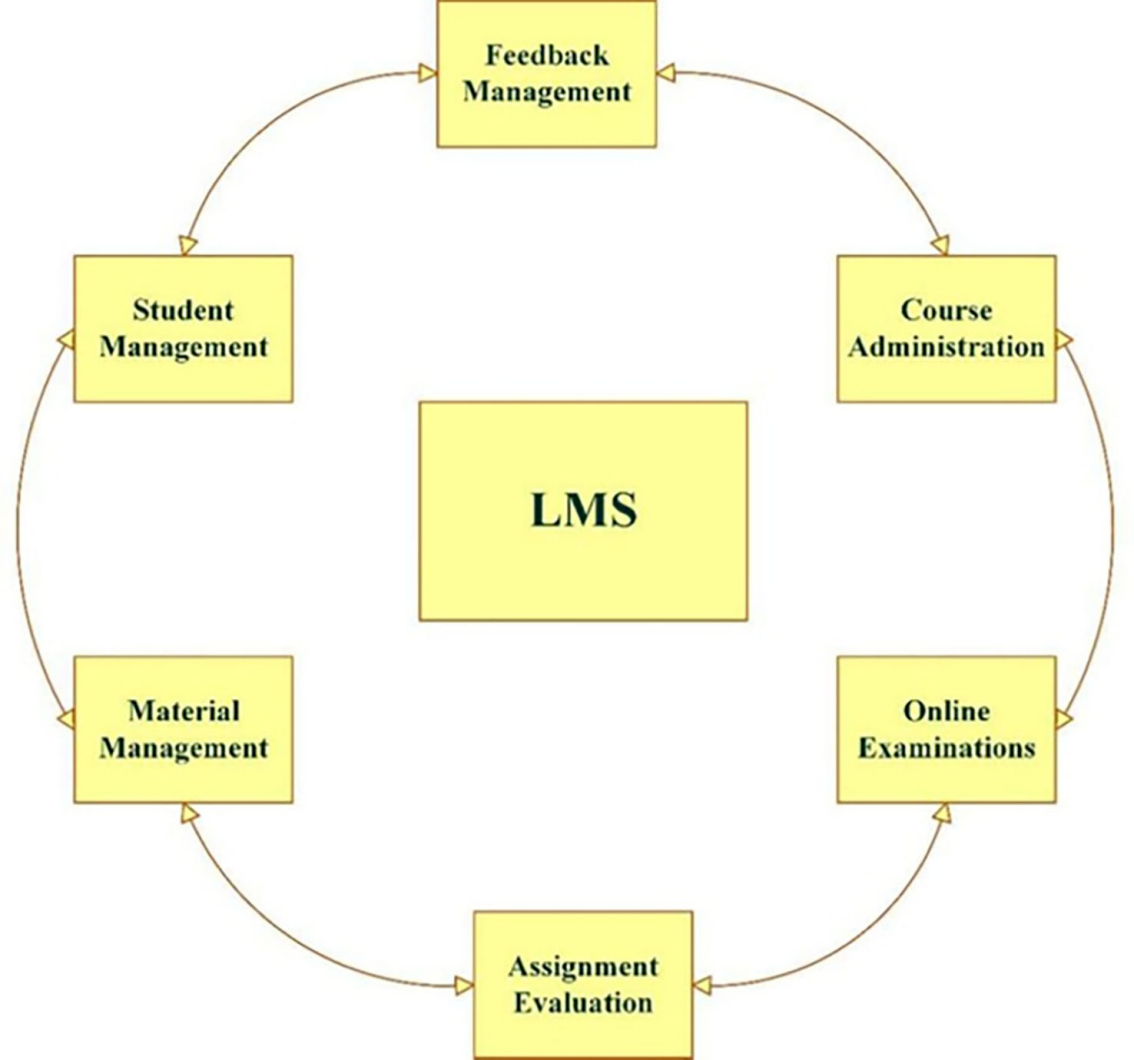
Learning management system ( Khalaf et al., 2022). A conceptual representation of the components and workflow within a learning management system (LMS).

In summary, collaborative learning styles and tools are crucial for improving e-learning experiences. Recent studies, ( Khalaf et al., 2022; Jena et al., 2023; Alanya-Beltran, 2024) highlight the benefits of integrating collaborative tools and adaptive technologies to foster more interactive and engaging educational settings. Adopting these strategies can enhance personalisation and effectiveness in learning for students. A Learning Management System (LMS) is a digital platform designed to manage, deliver, and track educational content and learning activities. These systems facilitate online learning by providing tools for content creation, communication, assessment and student progress tracking ( Saleh et al., 2024). As shown in Figure 2, the Learning Management System (LMS) plays a critical role in facilitating the integration of learning styles and collaborative filtering methods. By utilising LMS platforms, personalised learning experiences can be enhanced through real-time adaptation to student preferences and needs, leveraging models such as collaborative filtering. This aligns closely with the focus of this current study, which integrates collaborative filtering and learning styles to create a more personalised and collaborative learning environment. By bringing these elements together in an AI-driven system, the study aims to address the gaps in current learning management systems and improve both student engagement and academic performance in a more tailored way.

### 2.6 Personalisation of learning styles

The researcher from this study ( Sanjabi & Ali Montazer, 2020) explores the personalisation of e-learning environments using Kolb’s learning style model to enhance academic success and satisfaction. By tailoring learning strategies based on Kolb’s model, the paper evaluated the performance of students in a personalised e-learning course. Results demonstrated that students who received personalised learning experiences outperformed others in both academic success and satisfaction. While the model shows promise, the limitation lies in the need for further real-world testing and scalability analysis. The study by Sanjabi contributes to the development of adaptive learning systems by integrating Kolb’s learning styles.

The study by Sihombing et al. (2020) aimed to enhance e-learning systems by personalising content using the Felder-Silverman Learning Style Model (FSLSM). FSLSM categorises learning styles into four dimensions which are perception, processing, input, and understanding. Sihombing’s study digitised the Index of Learning Styles (ILS) questionnaire to identify users’ learning preferences and customised content to match these preferences. The results indicated that tailoring content based on FSLSM led to improved learner engagement and performance. However, the finding’s limitation was the small sample size, with only 30 students participating. This finding offers a valuable framework for advancing e-learning platforms by addressing individual learning preferences.

Furthermore, Rekha’s study ( Rekha et al., 2024) explores the use of AI in personalised learning environments, emphasising the impact on student engagement and performance. The study by Rekha highlights that AI-powered systems, such as adaptive learning paths, gamification, and predictive analytics, significantly enhance educational experiences by tailoring content to individual needs. Results show that these systems improve student engagement and performance by dynamically adjusting learning materials to each student’s pace and preferences. However, the study by Rekha notes a limitation in that personalised learning systems cannot guarantee a perfect fit for every student. Despite this, the finding provides valuable insights into how AI can support students in progressing according to the individual learning needs and effectively address learning gaps.

**Figure 3.**
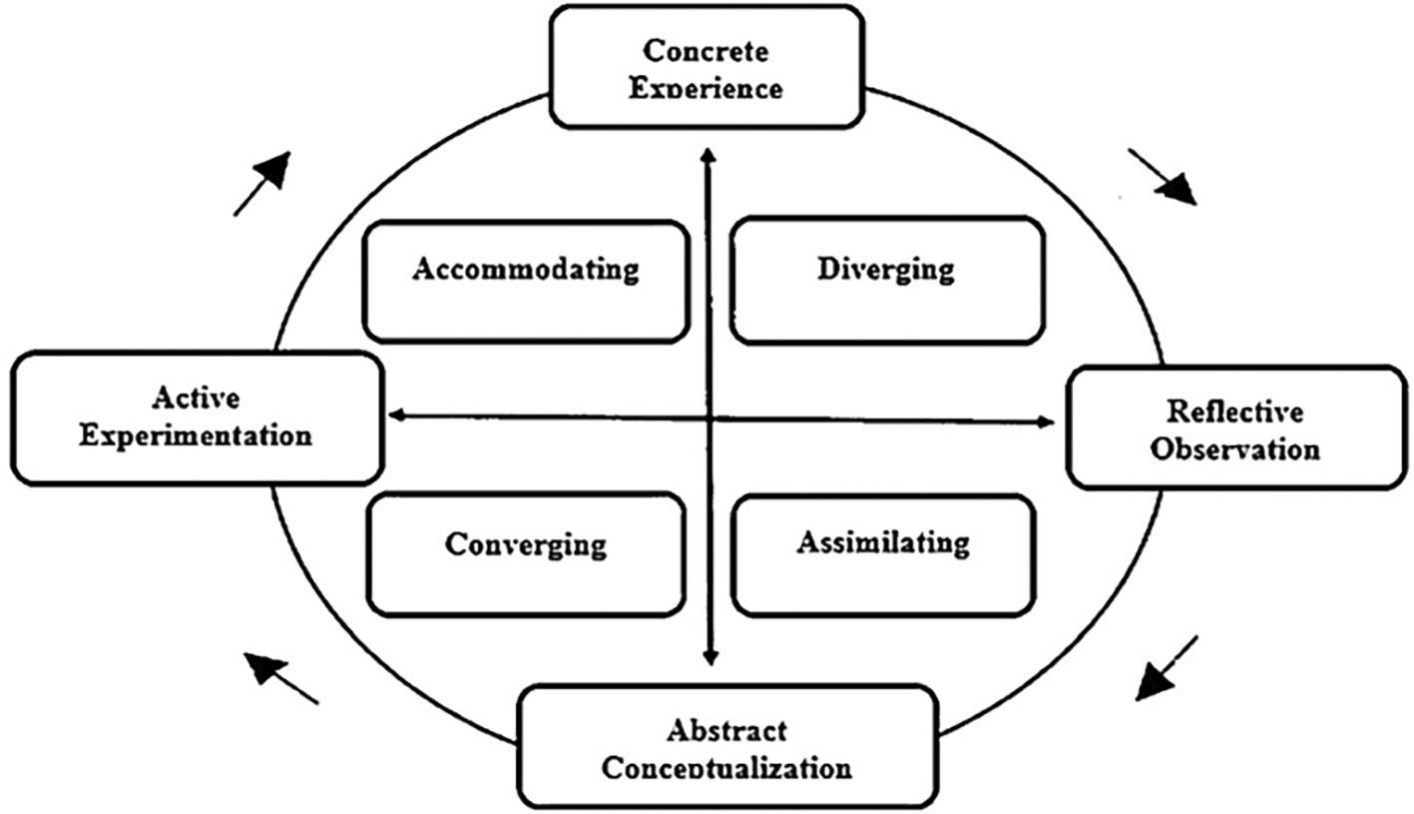
Kolb’s Learning Style model ( Sanjabi & Ali Montazer, 2020). The four-stage learning cycle and associated learning styles proposed by Kolb.

In conclusion, personalising e-learning environments through models like Kolb’s and Felder-Silverman enhances academic success and engagement. As shown in Figure 3, Kolb’s Learning Style Model provides a structured approach to categorising learning preferences, contributing to adaptive learning strategies. While Kolb’s model is widely referenced in personalised learning, this study focuses on AI-driven methods and collaborative filtering to improve adaptability in real-time, scalable e-learning environments. Kolb’s model provides useful categorisation of learning types but has limitations in real-time, scalable e-learning environments without the integration of other models. AI-driven systems further improve personalisation by adapting content to individual needs, although perfect alignment for every student remains a challenge. Overall, these approaches underscore the value of customisation in improving educational experiences.

In summary, this study explores learning style models and the application in education by reviewing various study sources. Various models are covered such as Kolb’s, Felder-Silverman, VARK, Gregorc’s, Riding’s, and Myers-Briggs and evaluate the impact on educational outcomes. The review includes detailed discussions on how personalised learning approaches and collaborative tools in e-learning platforms enhance academic performance and engagement.

## 3. Methods

**Figure 4.**
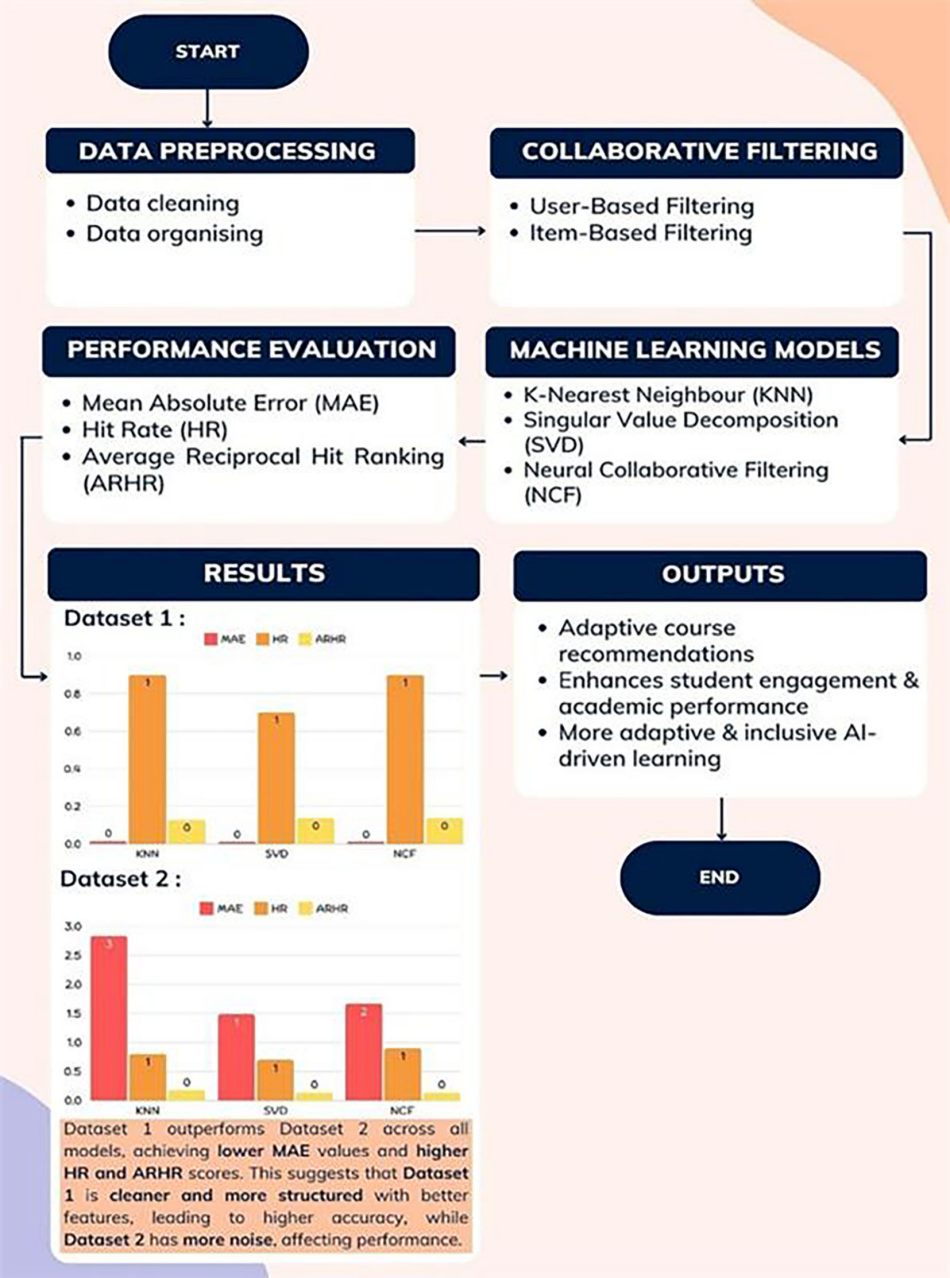
Proposed framework. The framework integrates collaborative filtering and machine learning to enhance e-learning outcomes.

This study employs the K-Nearest Neighbours (KNN) model, Singular Value Decomposition (SVD) model, and Neural Collaborative Filtering (NCF) model to implement recommendation and collaborative filtering mechanisms for e-learning course recommendations. Figure 4 presents the Proposed Framework, illustrating the integration of collaborative filtering and machine learning techniques within the AI-driven LMS. This framework outlines the process flow from data preprocessing to performance evaluation, enhancing recommendation accuracy and supporting an adaptive learning experience.

### 3.1 Dataset

The datasets that are used in this report are from the Kaggle website, which is a platform that allows users to find and publish datasets. The datasets that be chosen in this current study are education related ( Jena et al., 2023). The first dataset details the 100k scraped course reviews from the Coursera website as of May 2017. The second dataset is about the 209k courses detailed information and comments from the Udemy Courses website in Oct 2022. Both datasets have two files and are downloaded in a CSV file format.

The first dataset has 51,600 views and 6183 downloads, consisting of 140,321 data and 4 columns attributes. The second dataset has 17,800 views and 2,570 downloads, consisting of 209,735 data and 25 columns attributes.

The first dataset ( Adona, 2018) downloaded from Kaggle in the CSV file consists of 140,321 data and 4 columns, which are the following attributes: “Id”, “Course Id”, “Review” and “Label”. Table 2 presents the features in dataset 1.

**Table 2.** Features in dataset 1. This table describes the key features present in the Coursera dataset, including unique identifiers, review content, and labeled ratings.

No	Features	Description
1	Id	The unique identifier for a review.
2	Course Id	The course tag found in the URL of the course on the Coursera website.
3	Review	The actual text of the course review.
4	Label	The rating of the course review, categorised by score.

The second dataset ( Hossain, 2022) that downloaded consists of 209,735 data and 25 columns, which are the following attributes: “Comment Id”, “Course Id”, “Rate”, “Date”, “Name”, “Comment Text”, “Course Title”, “Course Type”, “Course Price”, “Course Headline”, “Number of Subscribers”, “Course Average Rate”, “Number of Reviews”, “Number of Comments”, “Number of Lectures”, “Course Content Length”, “Course Publish Date”, “Course Last Update”, “Course Category”, “Course Subcategory”, “Course Topic”, “Course Language”, “Course URL”, “Instructor Name” and “Instructor Profile URL”. Table 3 presents the features in dataset 2.

**Table 3.** Features in dataset 2. This table provides a description of features included in the Udemy dataset, such as course metadata, user comments, ratings, and instructor information.

No	Features	Description
1	Comment Id	The unique identifier assigned to each comment row.
2	Course Id	The unique identifier or tag for each course.
3	Rate	The numerical rating given by users for the course.
4	Date	The date associated with the comment.
5	Name	The name of the individual associated with the comment.
6	Comment Text	The actual text of the user’s comment on the course.
7	Course Title	The name of the course.
8	Course Type	Indicate whether the course is paid or free.
9	Course Price	The cost of the course listed on the platform.
10	Course Headline	A summary of the course.
11	Number of Subscribers	The total number of people enrolled on the course.
12	Course Average Rate	The average rating of the course based on user feedback.
13	Number of Reviews	The total number of reviews submitted for the course.
14	Number of Comments	The total number of comments about the course.
15	Number of Lectures	The total count of lectures included in the course.
16	Course Content Length	The total duration of the course content.
17	Course Publish Date	The date of the course was published.
18	Course Last Update	The date of the most recent update to the course content.
19	Course Category	The overarching category to which the course belongs.
20	Course Subcategory	The more specific classification of the course within the category.
21	Course Topic	The specific topic of the course.
22	Course Language	The language used for delivering the course content.
23	Course URL	The web link to access the course.
24	Instructor Name	The name of the instructor delivering the course.
25	Instructor Profile URL	The web link to the instructor’s profile on the platform.

Both datasets were obtained from Kaggle, a publicly accessible platform for sharing datasets. These resources are freely available under open licenses, and users can access the datasets by navigating to the dataset URLs provided in the Data Availability section. No sensitive personal information was included in the data. Preprocessing steps applied in this study, including cleaning and merging, can be reproduced using the original datasets as described in the Methods section.

### 3.2 Data preprocessing

**Figure 5.**
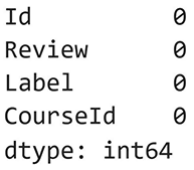
Check missing values for dataset 1. Visualisation showing missing data distribution in the Coursera dataset.

**Figure 6.**
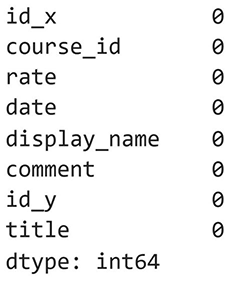
Check missing values for dataset 2. Visualisation showing missing data distribution in the Udemy dataset.

**Figure 7.**
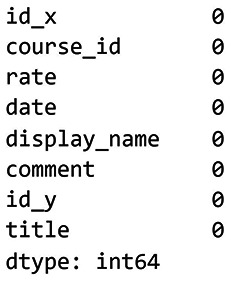
Remove missing values for dataset 2. Final version of the Udemy dataset after data cleaning.

After loading the dataset into Jupiter Notebook, then merge the two CSV files into one to combine relevant information from both sources. To ensure data integrity, duplicate entries were removed, maintaining a clean and unique dataset. Next, a comprehensive check for missing values was conducted and the drop function was employed to eliminate rows containing “Nan” values. Additionally, text preprocessing was performed on the “Review” column of both datasets to enhance the quality of the textual data. A custom function was applied to clean the reviews by removing special characters, extra whitespace, and converting the text to lowercase, ensuring consistency in the dataset. This cleaning step was essential for optimising the performance of subsequent text-based analysis and modeling. The sparsity of the resulting matrix was calculated, giving an indication of the proportion of missing values in the dataset. To improve computational efficiency, the matrix was converted into the Compressed Sparse Row (CSR) format, which is optimised for storing and processing sparse matrices. Finally, the dataset was split into training and testing subsets. This preprocessing stage ensured that the dataset was clean, consistent, and ready for the next steps of modeling and evaluation. Figure 5 shows the results of the missing values check for dataset 1. Figure 6 shows the results of the missing values check for dataset 2, while Figure 7 presents the results after removing the missing values from dataset 2.

### 3.3 K-Nearest Neighbours (KNN) model

**Figure 8.**
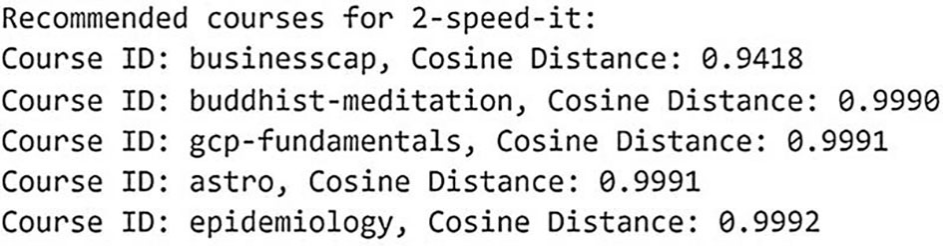
Recommended course in KNN model for dataset 1. Top course recommendations generated by the K-Nearest Neighbours model based on Coursera data.

**Figure 9.**
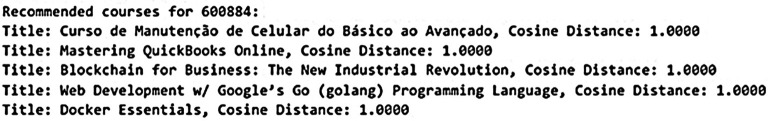
Recommended course in KNN model for dataset 2. Top course recommendations generated by the K-Nearest Neighbours model based on Udemy data.

After the data pre-processing step, this study involved implementing a K-Nearest Neighbours (KNN) algorithm to recommend courses based on user preferences ( Jena et al., 2023). The KNN model was chosen due to simplicity and effectiveness in collaborative filtering tasks. The KNN model was implemented using the scikit-learn library, with cosine similarity as the distance metric, commonly used in recommendation systems for measuring similarity between non-negative vectors. The model was trained using the sparse matrix of course-user interactions. A recommendation function was defined to retrieve the most similar courses based on similarity scores. Finally, the recommendations are presented along with the cosine distance, where a lower distance signifies higher similarity. The recommendations provide valuable insights into the course similarity patterns within the dataset. Figure 8 and Figure 9 below show the recommended course results in KNN model for dataset 1 and dataset 2.

### 3.4 Singular Value Decomposition (SVD) model

**Figure 10.**
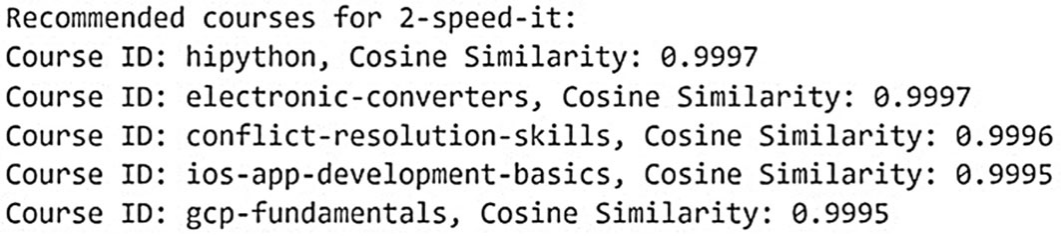
Recommended course in SVD model for dataset 1. Top course recommendations generated by the Singular Value Decomposition model based on Coursera data.

**Figure 11.**
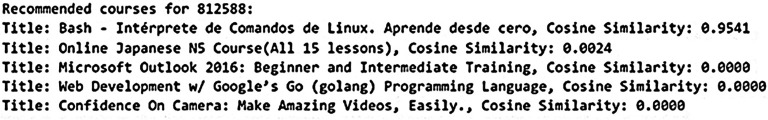
Recommended course in SVD model for dataset 2. Top course recommendations generated by the Singular Value Decomposition model based on Udemy data.

Besides that, the Singular Value Decomposition (SVD) model was used to recommend courses based on user preferences ( Jena et al., 2023). SVD is a technique that breaks down the user-item interaction matrix into smaller parts, helping to identify hidden factors influencing user choices. The SVD model was implemented using the Surprise library in Python with default settings. This allows the model to predict missing ratings or interactions, which is useful for recommending courses that users may be interested in. Figure 10 and Figure 11 below show the recommended course results in the SVD model for dataset 1 and dataset 2.

### 3.5 Neural Collaborative Filtering (NCF) model

**Figure 12.**
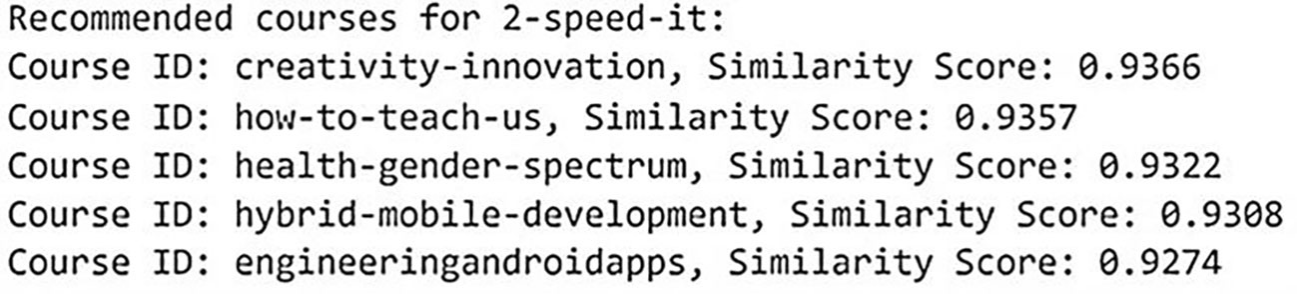
Recommended course in NCF model for dataset 1. Top course recommendations generated by the Neural Collaborative Filtering model for Coursera data.

**Figure 13.**
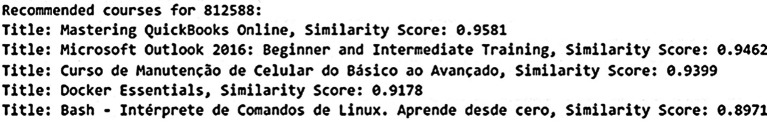
Recommended course in NCF model for dataset 2. Top course recommendations generated by the Neural Collaborative Filtering model for Udemy data.

Neural Collaborative Filtering (NCF) is a deep learning-based approach to collaborative filtering that uses neural networks to model user-item interactions ( Jena et al., 2023). Unlike traditional methods like KNN or SVD, which rely on linear relationships, NCF captures complex, non-linear interactions between users and items. In this study, the NCF model was implemented using Python with TensorFlow and Keras libraries. This approach is better suited for large and complex datasets. Figure 12 and Figure 13 below show the recommended course results in NCF model for dataset 1 and dataset 2.

### 3.6 Train-test split and model evaluation

To evaluate the performance of the recommendation model, the dataset was split into training and testing subsets using a 60-40 ratio, 70-30 ratio and 80-20 ratio ( Jena et al., 2023). The training data was used to train the KNN model, SVD model and NCF model. On the other hand, the test set contains unseen user-course interactions that the model has not encountered before. The model is trained on the training data and evaluated using the test data. The purpose of using test data is to simulate how well the model would perform in a real-world scenario, where it would need to predict recommendations for users based on unseen interactions. This helps in assessing the accuracy and effectiveness of the model in recommending courses that match user preferences.

### 3.7 Performance metrics calculation

The performance of the model was further analysed by refining the calculation of Mean Absolute Error (MAE), Hit Rate (HR), and Average Reciprocal Hit Ranking (ARHR) ( Jena et al., 2023). The “calculate_metrics” function was designed to compute these values by comparing the model’s recommendations with the actual ratings in the test dataset. Once the metrics were calculated, the results provided valuable insights into the model’s performance. The Mean Absolute Error (MAE) helped to assess the model’s overall accuracy, a lower MAE indicates better accuracy, as the predicted ratings are closer to the actual ratings. The Hit Rate (HR) measured how often relevant courses were recommended, a higher HR is better, as the model is successfully recommending relevant courses to users. The Average Reciprocal Hit Ranking (ARHR) evaluated the quality of the ranking, with a focus on how high the relevant courses appeared in the recommendation list, a higher ARHR indicates better ranking quality, as more weight is given to relevant courses that appear at the top of the list.

## 4. Results

### 4.1 K-Nearest Neighbours (KNN) model results

The KNN model was evaluated using three different training-test split ratios which are 60-40, 70-30, and 80-20, for both datasets 1 and 2. The performance metrics of Mean Absolute Error (MAE), Hit Rate (HR), and Average Reciprocal Hit Ranking (ARHR) were calculated for each split ratio to assess the effectiveness of the model. As the training data increased, the KNN model showed significant improvements in all metrics. The MAE decreased steadily, indicating more accurate predictions as more data was used for training. The HR increased, suggesting that a greater proportion of relevant courses were recommended. The ARHR remained consistent for the 70-30 and 80-20 splits, highlighting the ranking quality of the recommendations, and remained relatively stable with larger training data. For Dataset 2, the KNN model showed a declining performance with increasing training data. The MAE was much higher in Dataset 2 than in Dataset 1, reflecting the model’s lower predictive accuracy. The HR also decreased as the training data size grew, indicating that fewer relevant courses were being recommended in the larger training datasets. The ARHR showed similar trends, decreasing with larger training splits, suggesting that the model’s ranking of relevant courses became less effective as the training data increased. Tables 4, 5 and 6 show the performance metrics results for each ratio in dataset 1 and dataset 2.

**Table 4.** KNN model results for 60-40 ratio in dataset 1 and dataset 2. The performance of the K-Nearest Neighbours model is shown using Mean Absolute Error (MAE), Hit Rate (HR), and Average Reciprocal Hit Ranking (ARHR) for both datasets under 60:40 split.

	MAE	HR	ARHR
Dataset 1	0.0152	0.9000	0.1267
Dataset 2	2.8332	0.8000	0.1783

**Table 5.** KNN model results for 70-30 ratio in dataset 1 and dataset 2. The performance of the K-Nearest Neighbours model is shown using Mean Absolute Error (MAE), Hit Rate (HR), and Average Reciprocal Hit Ranking (ARHR) for both datasets under 70:30 split.

	MAE	HR	ARHR
Dataset 1	0.0131	0.9200	0.1500
Dataset 2	2.6253	0.6000	0.2044

**Table 6.** KNN model results for 80-20 ratio in dataset 1 and dataset 2. The performance of the K-Nearest Neighbours model is shown using Mean Absolute Error (MAE), Hit Rate (HR), and Average Reciprocal Hit Ranking (ARHR) for both datasets under 80:20 split.

	MAE	HR	ARHR
Dataset 1	0.0080	0.9600	0.1500
Dataset 2	2.3101	0.4000	0.1067

### 4.2 Singular Value Decomposition (SVD) model results

The SVD model was evaluated using the same three training-test split ratios 60-40, 70-30, and 80-20 for both datasets. For Dataset 1, the SVD model showed minimal variation across different training-test splits. The MAE values were consistently low, indicating good predictive accuracy. However, the HR remained constant at 0.7000, suggesting that the model consistently recommended relevant courses, regardless of the training data size. Similarly, the ARHR remained stable at 0.1370, which indicates that the quality of rankings did not change significantly with different split ratios. For Dataset 2, the MAE values were much higher compared to Dataset 1, indicating that the SVD model struggled with predicting ratings in this second dataset. Despite this, the HR remained constant at 0.7000, which means the model continued to suggest a similar proportion of relevant courses. Additionally, the ARHR remained unchanged at 0.1370, reflecting consistent ranking quality across all split ratios. The results for the Mean Absolute Error (MAE), Hit Rate (HR), and Average Reciprocal Hit Ranking (ARHR) are summarised in Tables 7, 8 and 9 below.

**Table 7.** SVD model results for 60-40 ratio in dataset 1 and dataset 2. The performance evaluation of Singular Value Decomposition using three metrics across both datasets for a 60:40 split.

	MAE	HR	ARHR
Dataset 1	0.0103	0.7000	0.1370
Dataset 2	1.4842	0.7000	0.1370

**Table 8.** SVD model results for 70-30 ratio in dataset 1 and dataset 2. The performance evaluation of Singular Value Decomposition using three metrics across both datasets for a 70:30 split.

	MAE	HR	ARHR
Dataset 1	0.0100	0.7000	0.1370
Dataset 2	1.4556	0.7000	0.1370

**Table 9.** SVD model results for 80-20 ratio in dataset 1 and dataset 2. The performance evaluation of Singular Value Decomposition using three metrics across both datasets for a 80:20 split.

	MAE	HR	ARHR
Dataset 1	0.0105	0.7000	0.1370
Dataset 2	1.4511	0.7000	0.1370

### 4.3 Neural Collaborative Filtering (NCF) model results

The NCF model was evaluated with the same three training-test split ratios 60-40, 70-30, and 80-20 for both datasets. For Dataset 1, the NCF model demonstrated stable performance across the different training-test splits. The MAE values were consistently low, reflecting good predictive accuracy. The HR was consistently high at 0.9000, indicating that the model recommended a high proportion of relevant courses. Similarly, the ARHR value remained unchanged at 0.1370, which signifies a consistent ranking quality regardless of the training-test split ratio. For Dataset 2, the MAE values were significantly higher compared to Dataset 1, indicating that the NCF model had greater difficulty in predicting ratings. However, the HR remained constant at 0.9000, suggesting that the model continued to recommend a high proportion of relevant courses. Furthermore, the ARHR remained stable at 0.1370, indicating that the quality of rankings was not impacted by the larger training datasets. Finally, the results for the Mean Absolute Error (MAE), Hit Rate (HR), and Average Reciprocal Hit Ranking (ARHR) are presented in Tables 10, 11 and 12 below.

**Table 10.** NCF model results for 60-40 ratio in dataset 1 and dataset 2. The comparison of Neural Collaborative Filtering model performance on Dataset 1 and Dataset 2 using 60:40 data split.

	MAE	HR	ARHR
Dataset 1	0.0115	0.9000	0.1370
Dataset 2	1.6667	0.9000	0.1370

**Table 11.** NCF model results for 70-30 ratio in dataset 1 and dataset 2. The comparison of Neural Collaborative Filtering model performance on Dataset 1 and Dataset 2 using 70:30 data split.

	MAE	HR	ARHR
Dataset 1	0.0121	0.9000	0.1370
Dataset 2	1.6667	0.9000	0.1370

**Table 12.** NCF model results for 80-20 ratio in dataset 1 and dataset 2. The comparison of Neural Collaborative Filtering model performance on Dataset 1 and Dataset 2 using 80:20 data split.

	MAE	HR	ARHR
Dataset 1	0.0085	0.9000	0.1370
Dataset 2	1.6667	0.9000	0.1370

### 4.4 Comparative analysis of KNN, SVD, and NCF models

In comparing the results from the KNN, SVD, and NCF models, several key observations emerged. KNN performed better with Dataset 1 compared to SVD and NCF, demonstrating the lowest MAE, highest HR, and stable ARHR values across larger training datasets. This indicates that KNN effectively captures patterns within Dataset 1, benefiting from increased training data. The model’s performance improved as more data was used, highlighting the effectiveness of Dataset 1 for recommendation tasks. SVD also performed well with Dataset 1, but the results showed minimal variation across different training-test splits, suggesting limited responsiveness to changes in data size. However, Dataset 2 posed more challenges for SVD, as indicated by higher MAE values, although HR and ARHR remained unchanged.

On the other hand, NCF exhibited consistent HR values at 0.9000 for both datasets, indicating good relevance in the recommendations. Despite this, the NCF model’s MAE was notably higher for Dataset 2, signalling lower predictive accuracy when compared to KNN and SVD. The ARHR remained constant, indicating stable ranking quality, even with the higher MAE. These findings suggest that while KNN adapts well to structured datasets, NCF maintains strong relevance in recommendations, and SVD exhibits stable but less flexible performance across different datasets.

Jena et al. (2023) found that KNN outperformed other models in recommendation accuracy and ranking, which aligns with the findings in Dataset 1 of this study. However, while Jena’s study focused on general course recommendations using collaborative filtering, this study evaluates the same method across two datasets, examining the impact of dataset characteristics on recommendation accuracy.

This study evaluated the predictive accuracy and ranking performance of KNN, SVD, and NCF in AI-driven learning environments by applying the author’s collaborative filtering method to two datasets. The results align with existing studies on collaborative filtering-based recommendations, highlighting the significance of dataset quality in optimising recommendation accuracy. While AI-driven recommendations are widely recognised for improving engagement, the effectiveness of collaborative filtering models can vary depending on dataset structure and user interaction patterns.

Unlike traditional collaborative filtering approaches that focus on a single dataset, this study provides a comparative analysis of model performance across different datasets, highlighting the impact of dataset characteristics on recommendation effectiveness. While previous study has demonstrated the success of collaborative filtering in course recommendations, limited studies have examined how dataset variability influences predictive accuracy and ranking performance. The findings contribute to the advancement of intelligent learning systems by emphasising the role of dataset structure in optimising adaptive e-learning recommendations.

## 5. Conclusions

In summary, this study primarily focused on exploring the development of a Personalised and Collaborative Learning Experience (PCLE) framework to enhance student engagement and academic performance in AI-driven learning environments. This study aimed (1) to determine the factors contributing to students’ academic performance in the context of AI-driven digital transformation. (2) To investigate the effects of a student’s learning style, academic records and intelligence agent assistance on personalised collaborative learning experience (PCLE) for students. (3) To develop a framework of PCLE for predicting and improving students’ engagement level and academic performance for students. (4) To evaluate the student’s engagement level and academic performance with the proposed PCLE framework for students.

The findings highlight the critical role of AI-driven collaborative learning strategies in today’s digital education landscape, especially in the post-COVID-19 era. This study adopted the author’s method for Dataset 1 and Dataset 2, focusing on collaborative filtering and machine learning models rather than a personalised learning style approach. The comparative analysis of machine learning models revealed that KNN achieved the best performance for Dataset 1, while SVD and NCF demonstrated stable predictive accuracy across different datasets. These results suggest that dataset characteristics significantly influence the effectiveness of recommendation models.

Acknowledging the limitations of this current study is crucial. The study primarily focused on a limited set of machines learning models, leaving room for future research to explore additional techniques that could enhance prediction accuracy and collaboration. Furthermore, the study was conducted with two datasets, and additional studies are necessary to evaluate the framework’s effectiveness in various learning contexts and across a broader range of student populations.

Overall, this study offers significant contributions to the field of AI-driven education by demonstrating the potential of collaborative filtering and machine learning in improving student engagement and academic performance. The PCLE framework provides valuable insights for educators, offering a foundation for future innovations in adaptive and inclusive learning systems. Findings suggest that further refinement of AI-driven recommendation techniques can enhance personalised and collaborative learning experiences, paving the way for more effective digital education environments. Despite the limitations, results contribute to the field of AI-driven education and encourage future study on expanding adaptive learning frameworks to accommodate a wider range of learners and educational contexts.

### Ethical considerations

This study did not involve direct interaction with human participants or animals, so ethical approval was not required.

## Data Availability

The datasets used in this study are publicly available and properly licensed for reuse:
• Coursera Course Reviews Dataset (100,000 reviews), available at Kaggle under Open Database License (ODbL): https://www.kaggle.com/datasets/septa97/100k-courseras-course-reviews-dataset
• Udemy Courses Dataset (209,000 courses and reviews), available at Kaggle under Creative Commons Attribution-NonCommercial-ShareAlike 4.0 International License (CC BY-NC-SA 4.0): https://www.kaggle.com/datasets/hossaingh/udemy-courses/data Both datasets do not contain any sensitive or personal information and comply with data sharing standards. No extended data such as questionnaires, consent forms, or interview guides were generated for this study. This study used publicly available datasets, and all data processing steps are described in the Methods section.
