# Supplementary material for: Personalised and Collaborative Learning Experience (PCLE) Framework for AI-driven Learning Management System (LMS)
Source: F1000Res. 2025 Aug 20;14:809. [Version 1] doi: 10.12688/f1000research.166248.1 (PMC12432497; doi:10.12688/f1000research.166248.1)
Supplement: Peer review history

# Reviewer report: Reviewer response for version 1

**DOI:** 10.5256/f1000research.183209.r407951

**Published:** 2025-09-12

**Authors:** A Shobana Devi

**Affiliations:** 1 Department of Data Science and Business Systems, SRM Institutte of Science and Technology, Chennai, Tamil Nadu, India

**Minor Comments**

1. **Methodological Transparency**
  - The paper introduces KNN, SVD, and NCF models but does not provide sufficient details on **hyperparameters, model architectures, or tuning strategies**.
  - Reproducibility is limited without explicit mention of training epochs, learning rates, batch sizes (for NCF), or number of neighbours (for KNN).
  - Recommendation: Provide a complete methodology section with model configuration details to ensure reproducibility.
2. **Dataset Limitations**
  - The study relies exclusively on **MOOC datasets (Coursera, Udemy)**. These may not fully represent the dynamics of institutional LMS environments.
  - Both datasets are from Kaggle, meaning they might suffer from **selection bias, incomplete metadata, and noise**.
  - Recommendation: Expand evaluation to institutional LMS data or additional MOOC datasets; include an analysis of dataset biases.
3. **Evaluation Metrics**
  - Current metrics (MAE, HR, ARHR) focus only on **accuracy and ranking**.
  - Key recommender system metrics such as **coverage, novelty, serendipity, and diversity** are absent, though these are crucial in educational contexts where exposure to varied content is beneficial.
  - Recommendation: Add multi-dimensional evaluation beyond accuracy to demonstrate educational value.
4. **Integration of Learning Styles**
  - While the introduction discusses VARK, Kolb, and other frameworks, **these are not integrated into the experimental design**.
  - The PCLE framework is proposed as personalised and collaborative, but the results section does not evaluate whether recommendations align with learning styles or improve engagement.
  - Recommendation: Either (a) conduct experiments incorporating learning style features into the models, or (b) reframe claims to avoid overstating integration with learning theories.
5. **Pedagogical Validation Missing**
  - The study evaluates models on predictive accuracy but **does not assess actual student engagement, satisfaction, or academic performance**.
  - Without real-world validation, it is difficult to conclude that the PCLE framework improves learning outcomes.
  - Recommendation: Conduct a user study or pilot deployment in a real LMS environment to connect computational metrics with educational impact.
6. **Comparative Baselines**
  - No baseline models (e.g., popularity-based, random recommender, or matrix factorisation) are reported for benchmarking.
  - This makes it difficult to assess the true performance gain of KNN, SVD, and NCF.
  - Recommendation: Include baseline comparisons to contextualise results.
7. **Clarity of Figures and Tables**

- Figures showing missing values and recommendations are helpful but lack **clear captions and interpretation**. Some tables are dense and could be simplified.
- **8. Consistency of Terminology**
- At times, the paper uses “personalisation” interchangeably with “recommendation.” These terms should be clearly distinguished.

Is the work clearly and accurately presented and does it cite the current literature?

Yes

If applicable, is the statistical analysis and its interpretation appropriate?

Yes

Are all the source data underlying the results available to ensure full reproducibility?

Yes

Is the study design appropriate and is the work technically sound?

Yes

Are the conclusions drawn adequately supported by the results?

Yes

Are sufficient details of methods and analysis provided to allow replication by others?

Yes

Reviewer Expertise:

Big Data Analytics, Machine Learning, Deep Learning and Artificial Intelligence

I confirm that I have read this submission and believe that I have an appropriate level of expertise to confirm that it is of an acceptable scientific standard.

# Reviewer report: Reviewer response for version 1

**DOI:** 10.5256/f1000research.183209.r407950

**Published:** 2025-09-08

**Authors:** Rifki Wijaya

**Affiliations:** 1 School of Computing, Telkom University, Bandung, West Java, Indonesia

The study demonstrates rigorous analysis and contains sufficient documentation to reproduce the results. As an additional analysis, the authors could perform NLP on the “Course review” field (e.g., lemmatization, term frequency–inverse document frequency) and visualize the most frequent terms using a word cloud. This would provide a concise graphical representation of prevalent student sentiments.

Is the work clearly and accurately presented and does it cite the current literature?

Yes

If applicable, is the statistical analysis and its interpretation appropriate?

Yes

Are all the source data underlying the results available to ensure full reproducibility?

Yes

Is the study design appropriate and is the work technically sound?

Yes

Are the conclusions drawn adequately supported by the results?

Yes

Are sufficient details of methods and analysis provided to allow replication by others?

Yes

Reviewer Expertise:

data science, Artificial intelligence, big data, biomedical engineering

I confirm that I have read this submission and believe that I have an appropriate level of expertise to confirm that it is of an acceptable scientific standard.

# Response

**Published:** 2025-09-08

**Authors:** Sook Ling Lew

**Affiliations:** Multimedia University, Malaysia

We would like to sincerely thank Dr. Rifki Wijaya for the constructive comments and approval of our publication. We greatly appreciate the valuable suggestions, which will help us further strengthen the study outcomes and enhance the overall contribution of our research.
